# Crescentic glomerulonephritis in children

**DOI:** 10.1007/s00467-019-04436-y

**Published:** 2020-02-12

**Authors:** Ulrike Mayer, Jessica Schmitz, Jan Hinrich Bräsen, Lars Pape

**Affiliations:** 1 Department of Pediatric Kidney, Liver and Metabolic Diseases, Hannover Medical School, Carl-Neuberg-Strasse 1, 30625 Hannover, Germany; 2 Department of Pathology, Nephropathology Unit, Hannover Medical School, Hannover, Germany

**Keywords:** Glomerulonephritis, Acute kidney injury, Prednisolone, Dialysis, Kidney biopsy, Children

## Abstract

**Background:**

To date, there is insufficient knowledge about crescentic glomerulonephritis (cGN), the most frequent immunologic cause of acute kidney injury in children.

**Methods:**

Over a period of 16 years, we retrospectively analyzed kidney biopsy results, the clinical course, and laboratory data in 60 pediatric patients diagnosed with cGN.

**Results:**

The underlying diseases were immune complex GN (*n* = 45/60, 75%), including IgA nephropathy (*n* = 19/45, 42%), lupus nephritis (*n* = 10/45, 22%), Henoch-Schoenlein purpura nephritis (*n* = 7/45, 16%) and post-infectious GN (*n* = 7/45, 16%), ANCA-associated pauci-immune GN (*n* = 10/60, 17%), and anti-glomerular basement-membrane GN (*n* = 1/60, 2%). Patient CKD stages at time of diagnosis and at a median of 362 days (range 237–425) were CKD I: *n* = 13/*n* = 29, CKD II: *n* = 15/*n* = 9, CKD III: *n* = 16/*n* = 7, CKD IV: *n* = 3/n = 3, CKD V: *n* = 13/*n* = 5. Course of cGN was different according to class of cGN, duration of disease from first clinical signs to diagnosis of cGN by biopsy, percentage of crescentic glomeruli, amount of tubular atrophy/interstitial fibrosis and necrosis on renal biopsy, gender, age, nephrotic syndrome, arterial hypertension, dialysis at presentation, and relapse. Forty-eight/60 children were treated with ≥ 5 (methyl-) prednisolone pulses and 53 patients received oral prednis(ol)one in combination with mycophenolate mofetil (*n* = 20), cyclosporine A (*n* = 20), and/or cyclophosphamide (*n* = 6), rituximab (*n* = 5), azathioprine (*n* = 2), tacrolimus (*n* = 1), and plasmapheresis/immunoadsorption (*n* = 5).

**Conclusions:**

The treatment success of cGN is dependent on early diagnosis and aggressive therapy, as well as on the percentage of crescentic glomeruli on renal biopsy and on the underlying type of cGN. CsA and MMF seem to be effective alternatives to cyclophosphamide.

## Introduction

**Fig. 1:**
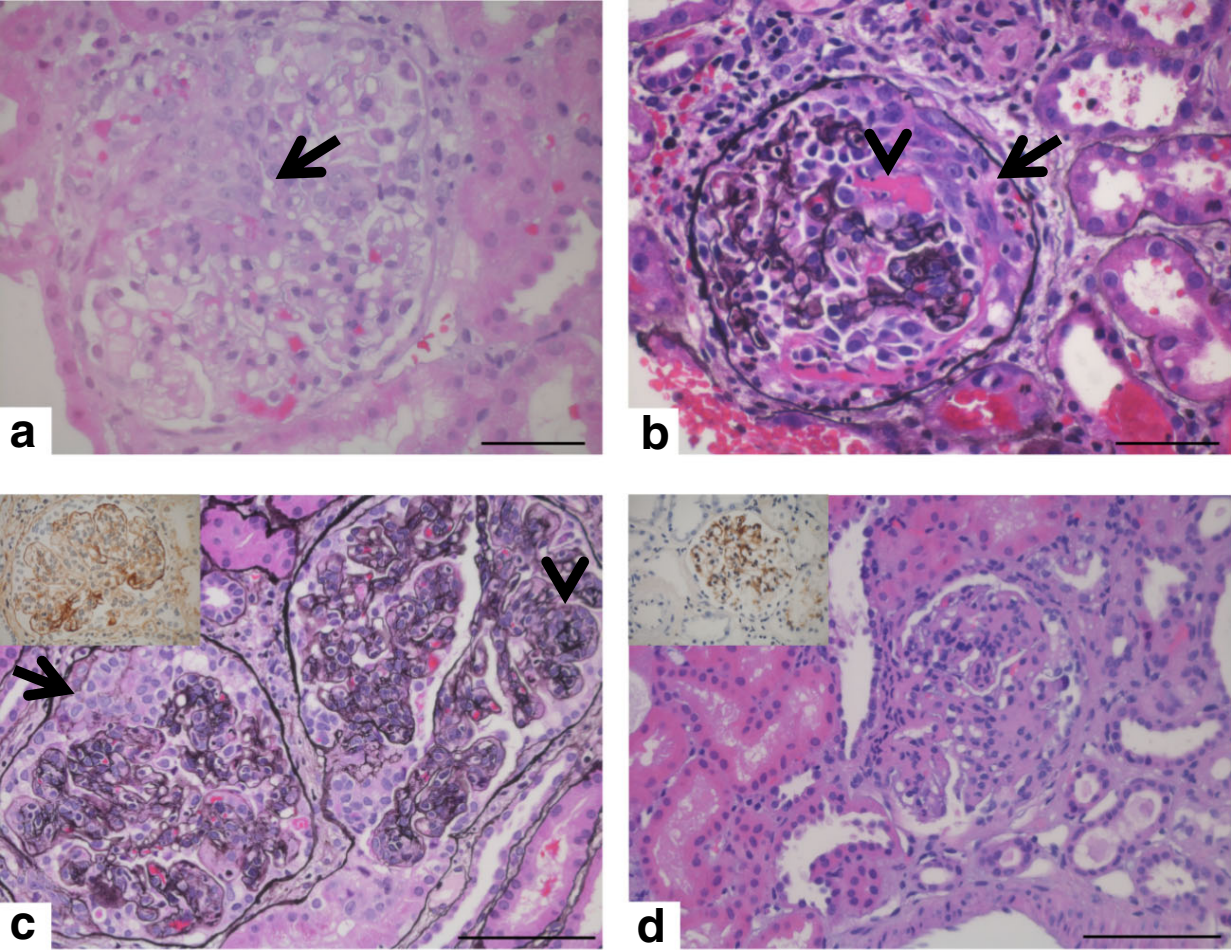
Histology of pediatric crescentic glomerulonephritis (cGN): **a** Granulomatosis with polyangiitis (GPA), female 16 years, arrow depicts cellular crescent. **b** ANCA-negative pauci-immune glomerulonephritis (GN), male 7 years; arrow points out cellular crescent and arrowhead fibrinoid necrosis. **c** Lupus nephritis revealing mesangiocapillary (arrowhead) and crescentic (arrow) proliferation (insert illustrates positive IgG immunohistochemistry (brown, DAB)), female 16 years. **d** Mesangioproliferative IgA nephropathy (insert shows glomerular positivity for IgA (brown, DAB), male 12 years. **a**, **d** H&E, **b**, **c** Jones methenamine counterstained with H&E. Bars represent 50 μm in **a**, **b**, and 100 μm in **c**, **d**.

Crescentic glomerulonephritis (cGN) is not a single disease entity but a pattern that can occur in a variety of glomerular diseases [1]. Caused by different pathomechanisms lesions and necrosis develop in glomerular capillaries in the case of systemic and kidney-restricted diseases. Ruptures of the glomerular basement membrane lead to fibrin exudation as well as cellular and humoral components of inflammation in the Bowman’s capsule. Parietal epithelial cells proliferate. This leads to the so-called extracapillary proliferations that narrow the remaining space in the capsule and appear as crescents on renal biopsy [1–3] (Fig. 1). Because of the ongoing inflammation, this process can lead to renal scarring. cGN is frequently associated with fast deterioration of kidney function and therefore often referred to as rapidly progressive glomerulonephritis (RPGN) [4]. Depending on the clinical context, the widespread definition of “crescentic GN” as a process involving > 50% of glomeruli [3] can be misleading, as there may be major diagnostic and clinical significance in the finding of even one fresh crescent.

The clinical course of cGN is dependent on the severity of the histopathological findings, i.e., by the percentage of glomeruli with crescents [5], but in adult series also on the underlying disease. In proliferative lupus nephritis, for example, the outcome is much worse than in post-streptococcal GN even if the percentage of glomeruli with crescents is similar at 25% [1]. An additional parameter for the prognosis is the severity of acute kidney injury at the time of diagnosis [2, 5].

The primary signs of cGN are hematuria, albuminuria, nephritic sediment, decreasing glomerular filtration rate (GFR), and oliguria. These renal symptoms might be associated with other organ manifestations in the case of an underlying systemic disease [3]. cGN is classified by different subtypes: Type I: anti-glomerular basement-membrane (anti-GBM) disease; Type II: GN caused by deposition of immune complexes (i.e., in IgA nephropathy (IgAN), lupus nephritis, post-infectious GN (PIGN), Henoch-Schoenlein purpura nephritis (HSPN)) and Type III: pauci-immune GN (i.e., caused by ANCA-vasculitis) [3]. In contrast to older patients in whom Type III predominates, Type II is the most common disease in the pediatric setting. Early induction therapy with intravenous (IV) steroids and/or cyclophosphamide is recommended in cGN, and in adults, plasmapheresis is sometimes used in addition [2]. In children, no such recommendations exist and adult guidelines are therefore used. However, the use of cyclophosphamide is declining, especially in children, because of long-term side effects such as infertility and carcinogenicity. Therefore, newer immunosuppressants such as cyclosporine A (CsA), mycophenolate mofetil (MMF), or rituximab have been evaluated [3]. Despite these general recommendations, the underlying disease has to be treated according to the disease-specific guidelines. As the 2012 Kidney Disease: Improving Global Outcomes (KIDGO) Guidelines are partly outdated and not child-specific, other published pediatric consensuses have to be followed.

Unfortunately, to date, only one publication on the clinical course and treatment of cGN has been published, nearly 30 years ago [6]. A more recent review of newer treatments and outcomes is therefore warranted. Consequently, we assessed the clinical course and morphological parameters of pediatric patients with biopsy-proven cGN in our center in order to determine diagnostic parameters for development of estimated glomerular filtration rate (eGFR) and dialysis-free survival.

## Patients and methods

Eight hundred eighty kidney biopsies performed in native and transplanted kidneys of children at Hannover Medical School between 1999 and 2015 were investigated, with crescents detected in 61/808 patients. Disease duration was defined as the time between first symptoms of the underlying disease and diagnoses of cGN on renal biopsy. For definition of crescents (cellular, fibrocellular and fibrous), the Oxford IgA classification was used [7]. A crescent was defined as extracapillary proliferation of more than two cell layers of any size (regarding the glomerular circumference); a cellular crescent was defined as > 50% of the proliferation occupied by cells, a fibrocellular crescent by < 50% of the lesion occupied by cells, and < 90% by matrix. Fibrous crescents (defined as > 90% of the lesion occupied by matrix) were not taken into account. Each patient with a minimum of one crescent in a biopsy was included in this analysis. A median of 20 glomeruli per biopsy (range 5 to 107) was evaluated. One 17-year-old patient was excluded because no follow-up data were available as he was treated in an adult nephrology unit, leaving 60 patients for analyses. All 60 kidney biopsies were re-evaluated by the same experienced nephropathologist (JHB) for this work using light, immunohistochemical, and electron microscopy. The pathologist was blinded to the patient’s data 12-month outcome. Clinical signs of GN, such as macrohematuria, edema, oliguria, arterial hypertension (defined by the Kinder- und Jugendgesundheitssurvey [Health Interview and Examination Survey for Children and Adolescents] (KiGGS) criteria [8]), non-renal signs and demographic parameters including age, gender, and duration of symptoms at presentation, were evaluated. Renal volume was measured by ultrasound (Ellipsoid formula) at time of diagnosis. At time of disease onset and at 1 week and after 1, 3, 6, and 12 months, the following laboratory values were documented: serum levels for creatinine, urea, albumin, electrolytes, hemoglobin, WBC, and eGFR as determined by the 2009 Schwartz bedside formula [9]. Arterial hypertension was defined as office blood pressures above the gender- and height-matched 95th percentile. End-stage renal disease (ESRD) was defined as need for renal-replacement therapy (dialysis, transplantation).

At time of diagnosis (time of renal biopsy), the following examinations and immunological parameters were determined: p-ANCA, c-ANCA, MPO-antibody (Ab) PR3-Ab, ANA, double-stranded-DNA Ab (anti-DNS), C3, C4, anti-streptolysin, IgA serum-level and glomerular basement-membrane Ab (anti-GMB) (Table 1(a)). As data evaluation was performed retrospectively, time ranges were used instead of exact time points and data was not available for each patient at each timepoint (Table 1(b)).

**Table 1:** Clinical tests performed and time points of evaluation

(a)						
Laboratory chemical tests						
*Serological investigations:* Creatinine, urea, albumin, protein						
*Immunological investigations:* P-ANCA, c-ANCA, MPO-Antibody (Ab) PR3-Ab, ANA, double-stranded-DNA Ab, C3, C4, anti-streptolysin, IgA serum-level and glomerular basement-membrane Ab.						
*Urine analysis:* Red blood cells and protein in urinary dipstick investigation, creatinine and albumin in spot urine.						
Clinical investigations Gender, age, weight, body length, blood pressure, disease duration, edema, oliguria, macrohematuria, treatment, relapse of disease during observation time..						
Renal ultrasound Measurement of kidney volume.						
Renal biopsy Light, immunohistochemical and electron microscopy.						
(b)						
Time point	T1	T2	T3	T4	T5	T6
Median time after kidney biopsy [days]	0	7	38	96	187	362
Range [days]		4–15	27–57	74–135	140–255	237–425
*N*	60	54	54	55	52	53

Data were documented using Microsoft Excel 365 (Microsoft Cooperation, Seattle, WA, USA). Time of disease duration was defined as the time between first clinical symptoms and kidney biopsy. Statistical analyses were performed with GraphPad Prism 5.0 (GraphPad, San Diego, CA, USA). Exploratory data analyses were primarily performed. All data was negatively tested for normal distribution. Therefore, median values between different groups were compared using the Mann-Whitney *U* test for pre-defined subgroups as gender, age, nephrotic syndrome, and arterial hypertension. Paired data was compared by Wilcoxon signed-rank test. Logistic regression analyses were performed to evaluate the relationship between one dependent binary variable and one or more nominal or ordinal independent variables. Kaplan Meier analyses were done to determine survival. *p* < 0.05 was considered as statistically significant. We have not based the analyses on the three categories of cGN, as there was only one patient in group I and as we could observe large differences between the underlying diagnoses in groups II and III in relation to outcome.

All patients have agreed with their hospital treatment contract that their data can be used for research in anonymized matter. The Ethics Committee of Hannover Medical School has agreed to this policy.

## Results

**Fig. 2:**
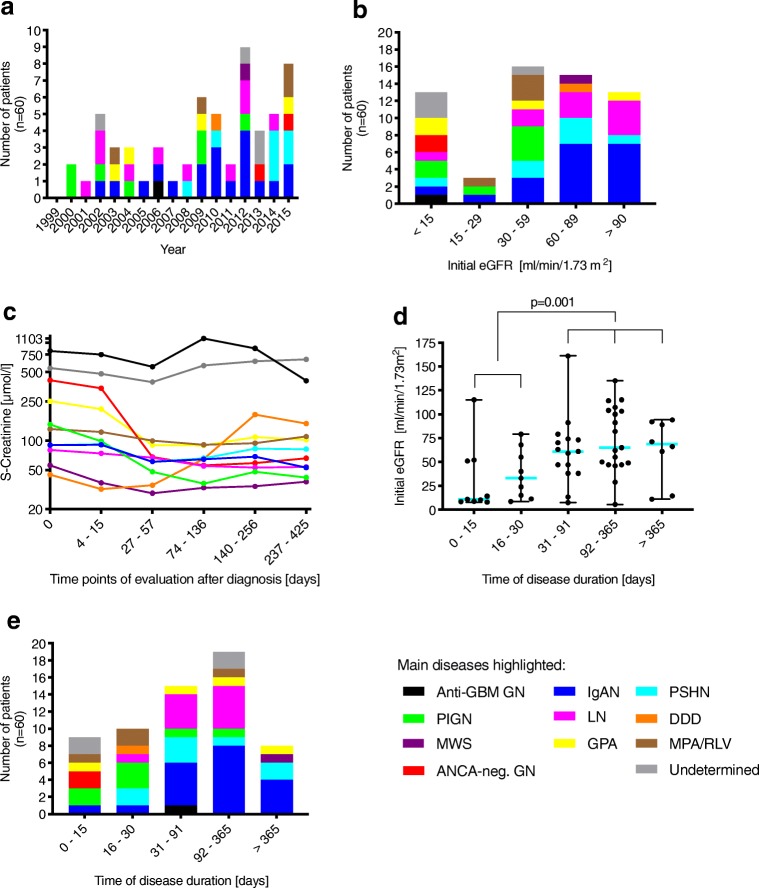
Number of crescentic glomerulonephritis (cGN) diagnosis per year and subclassification (**a**). Initial estimated glomerular filtration rate (eGFR) in the subgroups (**b**). Course of s-creatinine over the observation time (**c**). Initial eGFR depending on time of disease duration (**d**). Time of disease duration in the subgroups (**e**). *Abbreviations*: DDD, dense-deposit disease; GBM, glomerular basement-membrane; GPA, granulomatosis with polyangiitis; IgAN, IgA nephropathy; LN, lupus nephritis; MPA/RLV, microscopic polyangiitis and renal-limited vasculitis; MWS, Muckle-Wells Syndrome; PIGN, post-infectious glomerulonephritis; PSHN, Henoch-Schoenlein purpura nephritis

The 60 patients (median age 13 years, range 3–18, 31 male) could be subclassified into three groups of cGN and, further, by the underlying disease as demonstrated in the flow chart in Table 2 (which also gives the number of patients with data available 1 year after diagnosis). Interestingly, there was no gender difference overall. Figure 2 a shows the number of newly diagnosed diseases associated with cGN within the time period of the study. eGFR at time of diagnosis and courses of s-creatinine (serum-creatinine) in the disease groups are shown in Fig. 2b, c.

**Table 2:**
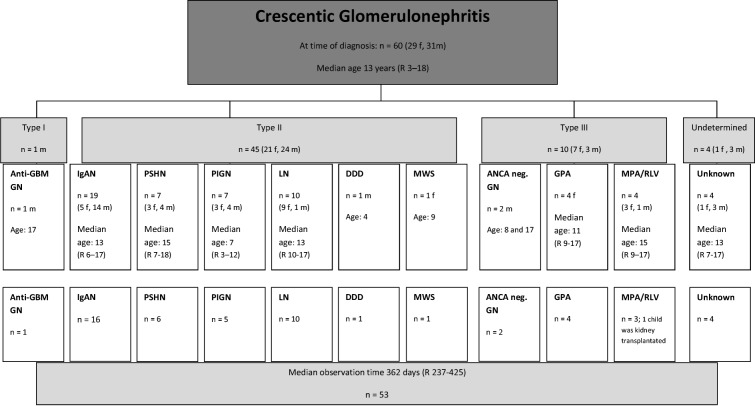
Subtyping and number of follow up of the patients with crescentic glomerulonephritis (cGN)

*DDD* dense-deposit disease, *GBM* glomerular basement-membrane, *GPA* granulomatosis with polyangiitis, *IgAN* IgA nephropathy, *LN* lupus nephritis, *MPA/RLV* microscopic polyangiitis and renal-limited vasculitis, *MWS* Muckle-Wells syndrome, *PIGN* post-infectious glomerulonephritis, *PSHN* Henoch-Schoenlein purpura nephritis

### Glomerular filtration rate

The median eGFR increased from 55 (range 4–161) from time of diagnosis to 92 ml/min/1.73m^2^ (range 5–175), *p* < 0.001, 1 year later, with differences in the three subgroups.

**Fig. 3:**
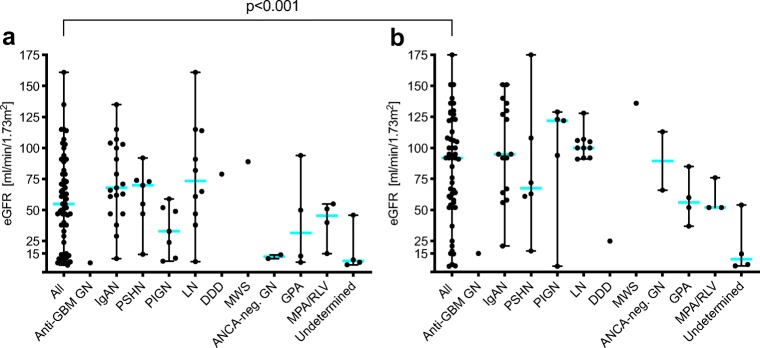
Course of estimated glomerular filtration rate (eGFR) during observation period dependent on underlying disease at time of diagnosis (**a**) and 1 year later (**b**). *Abbreviations*: DDD, dense-deposit disease; GBM GN, glomerular basement-membrane glomerulonephritis; GPA, granulomatosis with polyangiitis; IgAN, IgA nephropathy; LN, lupus nephritis; MPA/RLV, microscopic polyangiitis and renal-limited vasculitis; MWS, Muckle-Wells Syndrome; PIGN, post-infectious glomerulonephritis; PSHN, Henoch-Schoenlein purpura nephritis

The patient with cGN type I presented with terminal renal failure which did not significantly improve (eGFR 8 to 14 ml/min/1.73m^2^ at presentation and 1 year later, respectively). In children with cGN type II, median eGFR increased from 65 ml/min/1.73m^2^ (range 9–161) to 100 ml/min/1.73m^2^ (range 5–175), *p* < 0.001. In patients with cGN type III, median eGFR increased from 28 ml/min/1.73m^2^ (range 8–94) to 60 ml/min/1.73m^2^ (range 37–113), *p* = 0.013. Patients with a renal disease not classifiable by clinical and histopathological techniques had an initial median eGFR of 9 ml/min/1.73m^2^ (range 6–46) and a final median eGFR of 10 ml/min/1.73 m^2^ (range 5–54), *p* > 0.999 (Fig. 3a, b).

Patients with a fulminant progressive course of disease, defined as less than 30 days between time of first clinical symptoms and kidney biopsy (short disease duration) experienced a fast deterioration of kidney function (median eGFR ≤ 30 days 20 ml/min/1.73 m^2^ [range 8–115] and > 30 days 64 ml/min/1.73 m^2^ [range 6–161], *p* = 0.001) (Fig. 2d). They also presented with the highest urea values (median urea ≤ 30 days 20 mmol/l [range 4–40] and > 30 days 8 mmol/l [range 3–40], *p* < 0.001). GFR increased significantly during the observation time only in children with a disease course > 30 days (median eGFR disease course ≤ 30 days 57 ml/min/1.73m^2^ [range 5–151], *p* = 0.07 and disease course >30 days 94 ml/min/1.73 m^2^ [range 6–175], *p* = 0.003).

### Disease duration

The median duration time of the underlying disease before cGN was diagnosed in renal biopsy and therapy started was 60 days (range 3–1806). Patients with ANCA-negative pauci-immune GN demonstrated the shortest periods between diagnosis and therapy (3 to 5 days) as did the patients with microscopic polyangiitis and renal-limited vasculitis (MPA/RLV), PIGN, and dense-deposit disease (DDD). Patients with IgAN presented with a large variety in time between first symptoms and when biopsy was performed (range 22–1806 days) (Fig. 2e).

### Dialysis

**Fig. 4:**
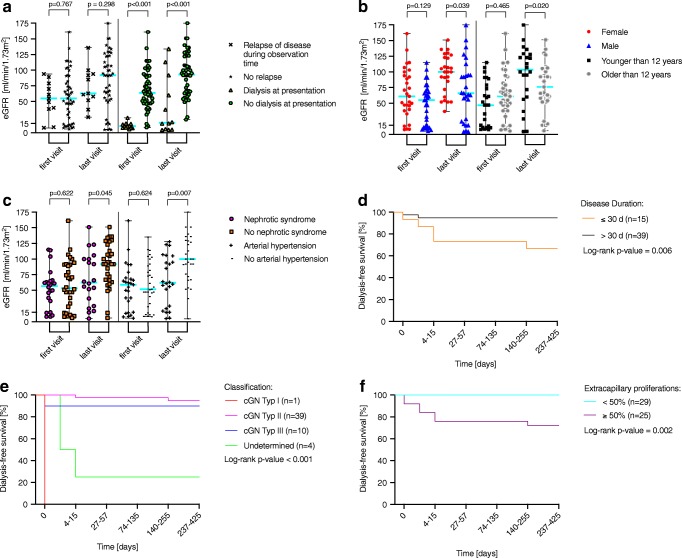
Factors influencing the course of estimated glomerular filtration rate (eGFR) during observation time. Disease relapse and dialysis at first visit (**a**), gender and age at time of disease onset (**b**), and nephrotic syndrome and arterial hypertension at time of disease onset (**c**). Kaplan-Meier curves of dialysis-free survival depending on disease duration (**d**), the classification of crescentic glomerulonephritis (cGN) (**e**), and the percentage of crescents (**f**)

Twelve children received initial dialysis (Table 3), with five remaining on dialysis during the observation period, one of whom received a kidney transplant. One patient required dialysis during the observation time without the need of dialysis at presentation. The need for primary dialysis was associated with a significantly worse outcome of kidney function: median eGFR primary dialysis 16 ml/min/1.73 m^2^ (range 5–134) versus no primary dialysis 93 ml/min/1.73 m^2^ (range 21–175), *p* < 0.001 (Fig. 4a).

**Table 3:** Clinical data of the total cohort

Pat.ID	eGFR [ml/min/1.73 m^2^]		Urinary dipstick analysis		Albumin-to-creatinine ratio [g/mol]		Glomeruli with crescents [% of all glomeruli]	Disease duration [days]	Methyl-/prednisolone pulse therapy (*n*: initial + during the course of observation)	Predniso-(lo)ne (*n*: initial + during the course of observation)	Additional immunosuppressive therapy (initial + during the course of observation)	Hemodialysis (HD), peritoneal dialysis (PD), plasma exchange (PE), immunoadsorption (IA) (initial + during the course of observation)
	First visit	Last visit	RBC [/μl]	Protein [mg/dl]	First visit	Last visit						
Anti-GBM glomerulonephritis												
1	8	HD 14	250	Neg.	15	Anuria	100	48	Yes (5)	Yes (ca. 50)	CYC 14 days p.o. + 1× RTX	HD, PE: 3 + 6
IgA nephropathy												
10	38	58	250	500	392	4	42	41	Yes (6 + 2)	Yes (219)	MMF	No
13	47	95	250	100	211	23	89	70	Yes (6)	Yes (42)	No	No
14	115	151	200	300	599	1	42	12	Yes (6)	Yes (71)	CsA	No
18	68	^a^*49*	NBD	NBD	709	^a^*3*	13	17	Yes (6)	Yes (> 186)	MMF + CsA on top	No
25	91	95	200	100	86	3	22	42	No	Yes (184)	+ CsA	No
27	100	151	200	> 300	80	18	8	244	No	No	No	No
28	71	130	200	> 300	25	16	10	1766	Yes (6)	Yes (56)	No	No
30	11	140	250	Neg.	NBD	1	8	423	Yes (6)	Yes (6)	No	No
36	66	64	200	> 300	521	14	50	1806	Yes (6)	No	MMF	No
40	47	67	200	> 300	16	2	7	362	Yes (6)	Yes (278)	MMF	No
44	104	NBD	80	> 300	601	NBD	6	194	Yes (6)	Yes (unknown)	CsA	No
47	29	92	200	> 300	77	4	31	309	Yes (5)	Yes (56)	CsA	No
50	107	123	250	30	67	23	20	97	No	No	No	No
52	63	^a^*66*	200	> 300	611	^a^*377*	28	33	Yes (6)	Yes (unknown)	CsA	No
59	79	92	250	100	84	1	18	34	Yes (6)	Yes (69)	CsA	No
63	135	127	10	100	NBD	9	4	116	No	Yes (132)	No	No
67	61	21	200	> 300	276	679	25	1319	Yes (6 + 5 before NBx)	Yes (43)	CsA (for 3 years) + MMF on top	No
68	62	56	50	500	918	81	71	203	Yes (6)	Yes (55)	TAC (Liver-Tx)	No
69	103	136	200	> 300	367	144	6	305	No	No	No	No
*Median* range	*68* 11–135	*95* 21–151	*200*	*> 300*	*276*	*12*	*20* 4–89	*194* 12–1806	*Y: 14; N: 5*	*Y: 15; N: 4*	*CsA: 6 + 2; MMF: 4 + 1; TAC: 1;* *None: 7*	*No*
Henoch-Schoenlein Purpura nephritis												
24	55	61	200	> 300	2193	1	10	22	Yes (6)	Yes (122)	CsA	With recurrence: PE: 5 and HD: 6
29	92	NBD	> 200	100	136	NBD	12	836	Yes (5 before NBx)	Yes (unknown)	CsA	No
33	74	108	200	> 300	277	15	42	42	Yes (3)	Yes (113)	No	No
48	70	63	NBD	NBD	214	8	31	36	Yes (6 + 6)	Yes (unknown)	CYC 3 Mo p.o. + CsA	No
53	73	175	200	> 300	578	NBD	33	70	Yes (3)	Yes (108)	No	No
57	14	17	200	> 300	328	7	83	784	Yes (6)	Yes (85)	CsA	HD for 2 months
62	47	72	200	100	167	5	65	117	Yes (6)	Yes (107)	CsA	No
*Median* range	*70* 14–92	*68* 17–175	*200*	*> 300*	*277*	*7*	*33* 10–83	*70* 22–836	*Y: 7*	*Y: 7*	*CsA: 4 + 1,* *CYC: 1* *None: 2*	*PE: 1; HD: 2*
Post-infectious glomerulonephritis												
8	52	129	200	> 300	246	4	13	8	No	No	No	No
11	49	94	200	> 300	242	2	50	101	No	Yes (149)	With recurrenceCsA	No
16	9	*134*	NBD	NBD	88	*2*	74	9	Yes (6)	Yes (60)	MMF	PD 9 d
22	33	122	200	> 300	361	3	50	25	Yes (3 + 2)	Yes (78)	No	No
23	59	123	200	> 300	736	128	67	32	Yes (6 + 11–13)	Yes (unknown)	No	No
32	24	*92*	50	500	969	NBD	21	20	Yes (6)	Yes (unknown)	No	PD 4 d
58	11	PD 5	250	500	NBD	Anuria	100	17	Yes (5 + 6)	Yes (63)	No	PD
*Median range*	*33* 9–59	*122* 5–129	*200*	*> 300*	*246*	*3*	*50* 13–100	*20* 8–101	*Y: 5; N: 2*	*Y: 6; N: 1*	*CsA: +1;MMF: 1;* *None: 5*	*PD: 3*
Lupus nephritis												
7	65	92	200	> 300	542	5	59	175	Yes (6)	Yes (291)	CsA	No
26	61	100	200	300	282	7	27	37	Yes (+ 6)	Yes (248)	CYC 4× IV + CsA	No
34	91	128	80	> 300	74	1	9	102	No	Yes (> 365)	MMF	No
41	9	92	250	30	214	77	97	27	Yes (6)	Yes (161)	MMF + 1× RTX	No
46	82	91	80	> 300	NBD	25	17	123	Yes (6)	Yes (193)	MMF	No
49	161	105	80	30	7,9	1	7	43	No	Yes (278)	MMF	No
55	114	100	200	> 300	539	11	29	192	Yes (6)	Yes (280)	CsA	No
56	47	100	250	500	481	5	18	40	Yes (6)	Yes (222)	MMF	No
65	115	107	200	> 300	75	1	9	173	Yes (6)	Yes (> 365)	CsA	No
66	38	106	200	> 300	915	40	63	74	No	Yes (> 365)	CYC 6x IV	No
*Median* range	*74* 9–161	*100* 91–128	*200*	*> 300*	*282*	*6*	*23* 7–97	*88* 27–192	*Y: 6 + 1; N: 3*	*Y: 10*	*CsA: 3 + 1; MMF: 5;* *CYC: 2;* *RTX: + 1*	*No*
Dense-deposit disease												
39	79	PD 25	NBD	NBD	6541	NBD	94	23	Yes (6 + 6)	Yes (63 + 89)	CsA	+ HD then PD
Muckle-Wells syndrome												
42	89	136	200	> 300	141	8	28	381	Yes (6)	Yes (161)	Ilaris	No
ANCA-negative vasculitis with pauci-immune glomerulonephritis												
35	10	113	200	> 300	238	*2*	100	5	Yes (6)	Yes (> 365)	MMF	HD: 2; IA: 5
51	14	66	> 200	100	38	1	50	3	Yes (6)	Yes (80)	MMF	PE: 6
Granulomatosis with polyangiitis with pauci-immune glomerulonephritis												
4	50	52	10	> 300	387	^*a*^*49*	50	330	Yes (4 before NBx)	Yes (unknown)	CYC p.o. (before NBx); AZA	No
6	13	60	250	30	84	163	76	50	Yes (6)	Yes (> 365)	MMF, 3 × RTX	PE: 3; PD initial; + HD 4 days
9	8	37	250	30	244	95	77	4	Yes (6 + 6 + unknown)	Yes (> 365)	+ CYC 1× IV, MMF, 1× RTX	PD 9 days
61	94	85	NBD	NBD	20	9	7	521	Yes (6 + 3 + 2)	Yes (unknown)	MMF + 4× RTX, AZA	No
*Median* range	*32* 8–94	*56* 37–85	*250*	*30*	*164*	*95*	*63* 7–77	*190* 4–521	*Y:3 + 1*	*Y: 4*	*MMF: 2 + 1; CYC: 1 + 1; RTX: 1 + 2; AZA: 1 + 1*	*PE: 1; PD: 2; HD: +1*
Microscopic polyangiitis and renal-limited vasculitis with pauci-immune glomerulonephritis												
5	40	52	NBD	NBD	NBD	13	88	26	Yes (6)	Yes (259)	MMF	No
43	51	52	200	100	167	^a^*16*	20	15	No	Yes (182)	MMF	No
54	55	76	NBD	NBD	76	4	64	363	Yes (6)	Yes (99)	MMF	No
60	15	*PD*^a^*5*	NBD	NBD	331	NBD	100	18	Yes (3 before NBx)	Yes (96)	No	HD then PD; NTx
*Median* Range	*46* 15–55	^a^*52* 5–76	*200*	*100*	*167*	*9*	*76* 20–100	*22* 15–363	*Y: 2 + 1; N:1*	*Y: 4*	*MMF: 3; None: 1*	*HD: 1; PD: + 1: NTx: 1*
Undetermined												
2	10	14	200	> 300	683	NBD	100	14	Yes (6 + 3)	Yes (ca. 163)	MMF intermittent	HD: 11 days
21	8	PD 5	200	> 300	432	Anuria	100	5	Yes (6)	No	No	PD
37	46	54	80	> 300	266	21	46	230	Yes (4)	Yes (unknown)	CsA	No
64	6	HD 6	200	> 300	1054	Anuria	80	98	No	No	No	HD
*Median* range	*9* 6–46	*10* 5–54	*200*	*> 300*	*558*	*21*	*90* 46–100	*56* 5–230	*Y: 3; N: 1*	*Y: 2; N: 2*	*CsA: 1;* *MMF: 1;* *None: 2*	*HD: 2; PD: 1*

*AZA* azathioprine, *CsA* cyclosporine A, *CYC* cyclophosphamide, *GBM* glomerular basement-membrane, *eGFR* estimated glomerular filtration rate, *IV* intravenous, *NBD* not determined, *MMF* mycophenolate mofetil, *N* no., *NBx* kidney biopsy, *Neg.* negative, *NTx* kidney transplantation, *Pat. ID* patient ID, *p.o.* per oral, *RBC* red blood cell, *RTX* rituximab, *TAC* tacrolimus, *Tx* transplantation, *Y* yes

^a^Last value collected in case no 1 year data was available

### Clinical factors

There was no difference between initial eGFR between children > 12 years and younger patients. However, gain of function (eGFR) was lower in the older children: median GFR > 12 years 72 ml/min/1.73 m^2^ (range 6–151) compared to < 12 years 107 ml/min/1.73m^2^ (range 5–175), *p* = 0.020 (Fig. 4b).

Male gender was associated with worse outcome despite gender-independent eGFR at timepoint of diagnosis: median eGFR at last visit was 66 ml/min/1.73m^2^ (range 5–175) in boys and 100 ml/min/1.73 m^2^ (range 37–151) in girls, respectively *p* = 0.039 (Fig. 4b).

We also observed that initially nephrotic children showed reduced recovery of kidney function compared to the remainder of the cohort although the initial eGFR of both groups was not significantly different: median eGFR of initially nephrotic and initially non-nephrotic children at last visit was 63 ml/min/1.73 m^2^ (range 5–151) and 92 ml/min/1.73 m^2^ (range 6–151), respectively, *p* = 0.045 (Fig. 4c).

Forty-five percent of patients had arterial hypertension at the time of diagnosis. Arterial hypertension at the time of diagnosis was another independent risk factor of having poorer kidney function after 1 year: median eGFR of patients having initial arterial hypertension was 62 ml/min/1.73 m^2^ (range 5–128) compared to children with no initial arterial hypertension, 100 ml/min/1.73 m^2^ (range 5–175), *p* = 0.006 (Fig. 4c).

### Recurrence

Eleven patients developed a recurrence of underlying disease during the observation period. The increase of eGFR was significantly lower in this group with a median eGFR of 63 ml/min/1.73m^2^ (range 25–163) compared to the non-relapsing children 92 ml/min/1.73m^2^ (range 5–175), *p* = 0.298 (Fig. 4a).

### Clinical signs

Dialysis-free survival of the native kidney was different in Kaplan Meier analysis between those patients with a short (≤ 30 days) or longer period (> 30 days) of time from first documented symptom to biopsy (*p* = 0.006, Fig. 4d), between the different underlying diagnoses (*p* < 0.001, Fig. 4e), as well as between patients with greater or fewer than 50% of glomeruli with crescents (*p* = 0.002, Fig. 4f). At time of diagnosis, all children presented with hematuria, 55% with macrohematuria. Fifty/52 patients had proteinuria, with 58% being nephrotic. The albumin-to-creatinine-ratio (ACR) varied significantly between the patients regardless of the underlying disease (Table 3) and decreased from 266 g/mol (range 8–6541) at time of diagnosis to 8 g/mol (range 1–679), *p* < 0.001, 1 year later. At time of diagnosis, the patients with an eGFR less than 90 ml/min/1.73 m^2^ (median ACR 277 g/mol, range 15–6541) had a higher ACR than those with an eGFR > 90 ml/min/1.73 m^2^ (median ACR 83 g/mol, range 8–601), *p* = 0.045.

Serum IgA was determined in 47 of the patients. Twenty-nine children presented with normal IgA values, 12 of them with IgAN and two with PSHN. From 18 children with increased IgA, two suffered from HSPN, six from IgAN and ten from other underlying diseases. Complement factor C3 was normal in 68% of cases. All children with lupus GN and DDD had decreased C3. P-ANCA and c-ANCA could be detected in ten and two children, respectively.

ANAs were negative in 27 patients and positive in all lupus nephritis children, whereas all lupus patients were also positive for anti-DNS. Anti-GBM-Ab was only positive in the single patient with anti-GBM GN. The anti-streptolysin titer was positive in 4/6 children with PIGN and in 11 children with other underlying diseases.

At time of diagnosis, renal ultrasound was only documented or available in 33/60 children. This is possibly due to the fact that many patients were not primarily seen at our center but transferred. Seventy percent presented with an elevated (> 95th percentile) and 18% with a borderline elevated (90-95th percentile) renal volume as compared to weight-matched normal values. Nephrotic range proteinuria was defined as a urinary albumin/creatinine ratio > 220 mg/mmol in spot urine.

### Pathology

Table 3 shows the results of the proportion of glomeruli with crescents (extracapillary proliferation) relating to the number of non-sclerotic glomeruli (median 38%, range 4 to 100). Fibrocellular crescents were found in only 21 patients, and they accounted for 19% of the total amount (80 fibrocellular versus 333 cellular crescents). In 26/60 patients, crescents were detected in ≥ 50% of the glomeruli. This criterion was fulfilled by 100% of the children with anti-GBM GN, DDD, and ANCA-negative pauci-immune GN, as well as in 75% of patients with systemic granulomatosis with polyangiitis (GPA), MPA/RLV, or unknown GN, and in 71% of children with PIGN. Only 16% of children with IgAN and 29% with HSPN had more than 50% crescents. This subgroup presented with a lower initial median eGFR (24 ml/min/1.73m^2^, range 6–79) compared to the patients with crescents in < 50% of glomeruli (74 ml/min/1.73 m^2^, range 11–161), *p* < 0.001. In addition, the improvement of eGFR was better for children with crescents in > 50% of the glomeruli (24 to 62 ml/min/1.73m^2^, range 5–123) compared to the other patients (74 to 100 ml/min/1.73m^2^, range 21–175), *p* < 0.001. In this context, it is important to refer to the 12 children with extracapillary proliferations in > 80% of glomeruli, as seven of these children developed ESRD, with initial median eGFR 11 ml/min/1.73 m^2^ (range 6–79) and final eGFR 17 ml/min/1.73 m^2^ (range 5–113), respectively, *p* = 0.3875. Nevertheless, three of the patients with extracapillary proliferations in > 80% of glomeruli, who initially experienced moderately impaired kidney function or kidney failure, had a normalization of eGFR within the observation period. It is also noticeable in this group that median duration of disease was 25 days (range 5–784) shorter than in the patients with extracapillary proliferations in < 80% of glomeruli. In regression analysis, the percentage of crescents correlated negatively with the eGFR at the end of observation time (R^2^ = 31%, *p* < 0.001) with more crescents leading to a lower eGFR.

Table 4 shows the additional renal biopsy findings from patients. Tubular atrophy and interstitial fibrosis of ≥ 20% and tubulointerstitial inflammation in ≥ 50% of the tubulointerstitium, as well as necrosis in ≥ 20% of glomeruli, were associated with a worse eGFR at last visit.

**Table 4:** Additional renal biopsy findings of patients

		Median eGFR [ml/min/1.73 m^2^] (range)	
		First visit	Last visit
Tubular atrophy/interstitial fibrosis	< 20% (*n* = 50)	55 (8–161)	94 (5–175)
	≥ 20% (n = 10)	56 (6–91)	54 (5–128)
	*pvalue*	*p = 0.425*	*p = 0.021*
Glomeruli with necrosis	< 20% (*n* = 49)	62 (6–161)	95 (5–175)
	≥ 20% (*n* = 11)	11 (8–70)	62 (5–113)
	*pvalue*	*p < 0.001*	*p = 0.022*
Tubulointerstitial inflammation	< 50% (*n* = 41)	65 (8–161)	95 (14–175)
	≥ 50% (*n* = 18)	24 (6–115)	58 (5–151)
	*pvalue*	*p < 0.001*	*p = 0.009*
Tubulointerstitial inflammation [Intensity 0–3]	0 (*n* = 1)	135	125
	1 (*n* = 20)	69 (9–161)	92 (21–151)
	2 (*n* = 28)	54 (8–115)	95 (5–175)
	3 (*n* = 10)	26 (6–82)	66 (5–123)
RBC casts [Intensity 0–3]	0 (*n* = 13)	50 (6–161)	85 (5–151)
	1 (*n* = 26)	64 (10–135)	100 (14–175)
	2 (n = 9)	70 (13–115)	67 (25–130)
	3 (*n* = 10)	11 (8–47)	92 (5–140)
Thrombi within glomeruli	Yes (*n* = 7)	13 (8–114)	78 (5–140)
	No (*n* = 51)	55 (6–161)	92 (5–175)
	*p value*	*p = 0.486*	*p = 0.62*
ATI severity score [Intensity 1–3]	1 (*n* = 7)	73 (47–161)	99 (52–175)
	2 (*n* = 32)	65 (6–115)	100 (6–151)
	3 (*n* = 21)	38 (8–82)	56 (5–113)
IgA [Intensity 0–3]	< 1 (*n* = 15)	50 (9–94)	66 (5–136)
	1 (*n* = 13)	63 (8–115)	92 (14–175)
	2 (*n* = 9)	59 (6–161)	99 (6–151)
	3 (*n* = 18)	70 (11–135)	100 (56–140)
IgG [Intensity 0–3]	< 1 (*n* = 15)	47 (6–135)	81 (6–151)
	1 (*n* = 23)	51 (9–115)	67 (5–175)
	2 (*n* = 12)	68 (9–114)	104 (21–129)
	3 (*n* = 5)	65 (47–161)	100 (92–105)
IgM [Intensity 0–3]	0 (*n* = 4)	32 (14–89)	59 (17–136)
	1 (*n* = 24)	67 (9–115)	98 (5–151)
	2 (*n* = 22)	57 (6–161)	60 (6–175)
	3 (*n* = 4)	47 (15–61)	100 (72–100)
C1q [Intensity 0–3]	0 (*n* = 5)	55 (13–94)	76 (60–136)
	1 (*n* = 15)	50 (9–115)	95 (5–151)
	2 (*n* = 19)	55 (8–107)	60 (17–175)
	3 (*n* = 18)	67 (6–161)	100 (6–128)
C3c [Intensity 0–3]	< 1 (*n* = 6)	53 (13–103)	71 (52–136)
	1 (*n* = 10)	70 (14–135)	92 (17–127)
	2 (*n* = 24)	64 (9–161)	92 (14–175)
	3 (*n* = 16)	50 (6–100)	107 (5–151)
Mesangial hypercellularity overall	≤ 50% of glomeruli (*n* = 23)	51 (8–103)	76 (5–136)
	> 50% of glomeruli (*n* = 32)	64 (6–161)	100 (6–175)
	*p value*	*p = 0.05*	*p = 0.09*
Mesangial hypercellularity global	≤ 50% of glomeruli (*n* = 29)	52 (8–161)	85 (5–136)
	> 50% of glomeruli (*n* = 26)	62 (6–135)	100 (6–175)
	*p value*	*p = 0.27*	*p = 0.18*
Mesangial hypercellularity segmental	≤ 50% of glomeruli (*n* = 53)	59 (6–135)	92 (5–175)
	> 50% of glomeruli (*n* = 2)	92 + 161	105
Intracapillary hypercellularity	Yes (*n* = 54)	57 (6–161)	92 (5–175)
	No (n = 2)	9 + 94	92 + 85
Intracapillary hypercellularity	≤ 50% of glomeruli (*n* = 35)	61 (8–161)	74 (5–175)
	> 50% of glomeruli (*n* = 21)	55 (6–115)	103 (6–151)
	*p value*	*p = 0.93*	*p = 0.16*

*eGFR* estimated glomerular filtration rate, *RBC* red blood cells, *ATI* acute tubular injury

### Treatment

Eighty percent of patients were treated with 5–6 (methyl-)prednisolone pulses with a median prednisolone dose of 309 mg/m^2^ body surface area (BSA) (range 281–513) if BSA was < 1.67 m^2^. The remaining patients were treated with 500 mg per day. The median dose for methylprednisolone was 561 mg/m^2^ BSA (range 275–708). In addition, 53 patients were treated with oral prednis(ol)one with a median duration of 151 days (range 6–> 365). Seventy-two percent of the total cohort was additionally treated with other immunosuppressants: MMF (*n* = 20), CsA (*n* = 20), cyclophosphamide (*n* = 6), azathioprine (*n* = 2), and rituximab (*n* = 5). One liver-transplanted patient continued with tacrolimus, and the patient the Muckle-Wells syndrome was additionally treated with canakinumab. Table 3 shows the mono- or combination therapy immunosuppressive treatments used in association with the underlying disease. Depending on the underlying disease, four patients received therapeutic plasmapheresis or immunoadsorption at time of disease manifestation. One patient with a relapse was treated with plasmapheresis as rescue therapy (Table 3).

## Discussion

This analysis of pediatric patients with cGN in our cohort revealed a wide inter-individual variability in initial kidney function which was independent of the underlying disease. An early treatment with IV(methyl-)prednisolone followed by oral steroids in combination with other immunosuppressants was most often successful. Outcome was dependent on percentage of glomerular crescents, disease duration, and the underlying type of cGN. No patient died during the observation time.

The frequency of underlying diagnoses is in accordance with the published literature [10], with the highest prevalence of immune complex crescentic GN. Regarding the percentage of crescents, the highest incidence in the total cohort was for IgAN. PIGN was most frequently associated with crescents in more than 50% of glomeruli.

Our results confirm other studies demonstrating that the percentage of glomeruli with crescents correlates with the severity of cGN [3, 5]. All of our seven patients who progressed to ESRD had crescents in more than 80% of the glomeruli, in most cases, a short disease duration and nearly all presented with acute kidney failure. However, even in the majority of patients with a high number of crescents, our results suggest that intensive immunosuppressive treatment has a good chance of success. However, our retrospective data analysis does not allow us to build a predictive model based on these factors. Only one patient required dialysis during the observation time. Another prognostic factor is the time of disease duration: those patients with a faster, more fulminant course of disease showed minor improvement in renal function and resulted in a higher percentage of ESRD. The term “rapid progressive glomerulonephritis”—though not clearly defined in the literature—can be used to describe this latter group. On the other hand, some patients (i.e., with IgA Nephropathy) had a long time between primary diagnosis of their underlying disease and detection of cGN on renal biopsy. For those patients, continuous, close monitoring of renal function seems to be important so that a sudden deterioration of their kidney function can be diagnosed early enough for successful intervention.

Interestingly, in addition to children with initial dialysis or disease relapse during observation, male patients, children with arterial hypertension or nephrotic syndrome at presentation, and those older than 12 years had a worse outcome. Further renal damage factors, such as tubular atrophy and interstitial fibrosis, glomerular fibrinoid necrosis, or tubulointerstitial inflammation, were associated with worse kidney function outcome.

Our results show that high kidney volume seems to be a good non-invasive surrogate parameter for the diagnosis of cGN, especially in combination with high blood pressure and urinary dipstick analysis positive for erythrocytes and protein.

Serum IgA did not prove itself as a marker for IgAN in our cohort, due to its low positive predictive value [11], which contradicts results published elsewhere [12]. The same is true for ASL-titer. In contrast, a decreased serum C3 was a good marker for lupus GN or DDD. On the other hand, as shown before, the anti-DNS titer did not correlate with disease severity of lupus GN [13].

MPO antibodies (p-ANCA) were detectable in all children with MPA/RLV, whereas in children with GPA, the detection of PR3 antibodies (c-ANCA) dominated, which broadly corresponds to the literature [14].

Historically, despite steroids, the primary immunosuppressant administered in cGN has been cyclophosphamide [13, 15–17]. As cyclophosphamide therapy is associated with a long-term risk for infertility and has a pro-oncogenic character, other immunosuppressive therapies such as CsA, MMF, and rituximab have been administered in many patients with good results (although treatment is obviously dependent on the underlying disease). This is especially important in children who have a long life expectancy. However, a detailed disease-based analysis of the efficacy of different immunosuppressive therapies is not possible in our cohort because of its heterogeneous, retrospective nature and the small number of patients in each group. The CD20 antibody rituximab has been shown to be effective in children with recurrence; however, we could not analyze the effect of rituximab in detail because of the large differences between the five rituximab patients in underlying diagnosis and disease course. In severe, antibody-based cGN, plasma exchange, or immunoadsorption were effectively used for induction therapy or in the case of a relapse.

Our analysis has several limitations. Because of the retrospective character of the analysis, patient clinical data were not documented in a standardized fashion. There are only a few uniformly accepted treatment recommendations for subdiagnoses of cGN in children, and over the long inclusion time of our analysis, these have changed. According to the high number of different underlying diagnoses, an analysis for differences in the relationship of all subdiagnoses and eGFR development was not statistically possible. Also, the influence of additional treatment strategies for the underlying diseases could not completely be analyzed. The same is true for side effects of medications, as the retrospective nature of this study did not allow a complete documentation of adverse events, especially when patients were seen by other physicians. The initial pathological evaluations on which therapeutic interventions were based were performed by different pathologists. Moreover, classifications and classification criteria have changed during the study period. Aiming at harmonization of morphological analyses, all histologies have been re-evaluated using accepted criteria by the same pathologist. Some patients had biopsies with fewer glomeruli than required by the Oxford Classification (8 glomeruli) or the HSPN criteria (10 glomeruli) [7, 17]. Interestingly, treatment of cGN in our center did not really change during the long observation time of our study. Steroid pulse therapy was consequently used over the whole period. Rituximab was introduced in our center for children as early as 2005. An exception was the use of cyclophosphamide, only administered in the earlier period until 2006 for some cases of lupus nephritis, GPA, and anti-GBM nephritis, according to older recommendations. Despite these mentioned limitations, this is the first analysis of a large group of children with cGN using a standardized pathological assessment by the same pathologist. Although some of the morphological data confirm published results of smaller collectives, a comprehensive morphological workup proves to be valuable in assessment of disease severity and therapeutic strategy planning.

We conclude that early detection and immediate aggressive treatment of cGN in children leads to stable remissions in the majority of patients and should therefore be implemented in most cases. To further determine more detailed therapy recommendations (including new, emerging therapies for cGN, such as mifepristone, budesonide or erlotinib [18–20] not yet used in children), improved cGN classification should be developed, including documentation of comprehensive clinical data in an international registry. Due to the small, heterogeneous group of patients, randomized, controlled trials do not appear to be feasible.
